# Survival, Chemotherapy and Chemosensitivity Predicted by CTC Cultured *In Vitro* of SCLC Patients

**DOI:** 10.3389/fonc.2021.683318

**Published:** 2021-06-25

**Authors:** Lixia Ju, Juan Yang, Changyun Zhai, Shuizhen Chai, Zhiyi Dong, Minghua Li

**Affiliations:** ^1^ Department of Integrative Medicine, Shanghai Pulmonary Hospital, Tongji University, Shanghai, China; ^2^ Department of Medical Oncology, Yancheng Traditional Chinese Medicine (TCM) Hospital, Nanjing University of Traditional Chinese Medicine, Yancheng, China; ^3^ Department of Oncology, Shanghai Municipal Hospital of Traditional Chinese Medicine, Shanghai University of Traditional Chinese Medicine, Shanghai, China

**Keywords:** CTC, survival, chemotherapy, chemosensitivity, small cell lung cancer

## Abstract

**Purpose:**

The prognosis for small cell lung cancer (SCLC) patients receiving later-line treatment is very poor and there is still no standard treatments after the second-line setting. Analyzing the susceptibility of chemotherapeutic drugs with circulating tumor cells (CTCs) cultured *in vitro* may contribute to optimize the therapeutic regimen. However, so far CTCs have been barely used for studying their chemosensitivity due to the lack of technology to obtain wholly intact and viable CTCs.

**Methods:**

Based on a retrospective study of the therapeutic response of 99 patients with unresectable SCLC, the CTC count in 14 SCLC patients was detected before and after chemotherapy to evaluate its role as a potential marker of response. Furthermore, the drug susceptibility of CTCs cultured *in vitro* obtained from ClearCell FX^®^ System was tested and the therapy response was evaluated.

**Results:**

All of the 99 patients received the first-line chemotherapy and the objective response rate (ORR) was 74.7%. A total of 36 patients received the second-line therapy and the average duration was 2.6 months, and only 11 cases out of them received the third-line therapy but no one responded. The change of CTC counts was identified to be correlated with therapy response. However all the five SCLC patients who were administered with the drugs according to the drug susceptibility test of CTCs for two cycles underwent progression of disease.

**Conclusion:**

The results showed that the responses of chemotherapy are very poor in later lines and the drug susceptibility test using CTCs primary cultured *in vitro* may not benefit the improvement of therapeutic regimen of SCLC patients.

## Introduction

According to the statistics, there would be 2.2 million new lung cancer cases in 2020, 15-20% of which were SCLC (1). SCLC is a highly aggressive malignancy and frequently with distant metastases at diagnosis. It is staged using the Veterans Administration Lung Study Group (VALSG) staging system, which divides SCLC patients into limited-stage (LS) diseases or extensive-stage (ES) diseases. And according to this staging system, almost two-thirds of patients have ES diseases at diagnosis (2). Even though SCLC is highly sensitive to initial chemotherapy and radiotherapy, the outcomes of newly diagnosed ES-SCLC patients are still very poor, with median progression-free survival (PFS) only about 5-6 months, median overall survival (OS) less than 10 months (2, 3). The reason for such poor survival of these patients is that the drug resistance to first-line chemotherapy emerged very quickly and the efficacy of second-line and subsequent therapies is undesirable. The standard treatment for newly diagnosed ES-SCLC at present is platinum-based doublet chemotherapy consisting of cisplatin or carboplatin plus etoposide or irinotecan alone or in combined with PD-L1 Inhibitors, and the response rates of the first-line chemotherapy are 60% to 85% (4, 5). Nevertheless, most of these patients quickly become resistant to these drugs, with a median PFS of 4 to 7 months (6–8).

Topotecan is the only Grade I recommended chemotherapeutic drug in second-line therapy for recurrent SCLC patients. There is no standard therapy for those patients eventually progressed on second-line chemotherapy. Palliative care/best supportive care (BSC) or other systemic chemotherapy can be the alternative option based on patient’s performance status. Some new drugs, such as immunotherapy agents (Nivolumab and Pembrolizumab) and the multi-targeting tyrosine kinase inhibitor Anlotinib, have been approved by Chinese Food and Drug Administration (CFDA) and are already available to physicians in China, but their survival benefits are very limited and most patients cannot afford these drugs which are not included in the coverage of medical insurance reimbursement.

CTCs are tumor cells that shed from primary and metastatic sites and circulate in the peripheral blood and can be detected by many advanced technologies. Hou et al. reported that CTCs were present in 85% of SCLC patients with the abundance of 1,589 ± 5,565 cells/7.5 mL blood (9). Huang et al. found that the median number of CTCs in 24 patients measured at baseline and post-treatment was 75 (range 0-3430) and 2 (range 0-526), respectively; the median reduction of CTCs from baseline to post-treatment was 97.4% in 15 subjects (10).

As there is no standard care and the prognosis and outcomes of SCLC patients are very poor in later lines of therapy, the precision treatment holds great promise for cancer patients. With the potential to address challenges associated with drug susceptibility and the variability among the patients, analyzing the susceptibility of chemotherapeutic drugs with primary cultured CTCs *in vitro* may provide some useful information for optimizing the therapeutic regimen and prolong survival time of SCLC patients. So far CTC has been barely studied for its chemosensitivity due to the lack of technology for obtaining wholly intact and viable CTCs. In this study, the ClearCell FX System was used to get intact and viable CTCs and then the CTCs were primary cultured *in vitro* and employed for investigations of their drug sensitivity profiles.

## Materials and Methods

### Patients Data

We retrospectively reviewed the medical records of patients with unresectable SCLC treated at Blinded for peer review between January 2014 and December 2019. All patients displayed measurable disease by the Response Evaluation Criteria in Solid Tumors (RECIST, Version 1.1) and an Eastern Cooperative Oncology Group (ECOG) performance status (PS) of less than or equal to 2. The information were collected including age, gender, laboratory results, diagnoses, stage, anatomic sites of involvement, sites of metastases, treatment plan, specific therapy, other medications such as supportive care agents, and performance status. The therapy response was evaluated after the two cycles of chemotherapy and every two subsequent cycles after the first evaluation until the disease progressed, and the results were recorded as complete response (CR), partial response (PR), stable disease (SD), and progressive disease (PD) according to RECIST 1.1 criteria.

### Study Design

These studies were prospective single-institution clinical studies conducted at the Blinded for peer review. Patients aged ≥18 years with histologically or cytologically confirmed unresectable SCLC were enrolled. The therapy response was evaluated after the two cycles of chemotherapy and every two subsequent months after the first evaluation until the disease progressed, and was recorded as CR, PR,SD, and PD according to RECIST 1.1 criteria. The ORR was defined as the sum of CR plus PR. The disease control rate (DCR) was defined as the sum of CR plus PR plus SD. The treatment response was evaluated by CT scan two months after the initiation of chemotherapy and then every two months. Our study was approved by The Ethics Committee of Blinded for peer review (approval no. K19-137). All patients provided written informed consent prior to enrollment in the study.

### Monitoring of CTC Counts During Chemotherapy

In this study, patients were administrated with the first-line carboplatin plus etoposide chemotherapy and some of them were screened for CTC counts test using folate receptor targeted PCR by GENO Biology in China, within one week before and after two cycles of chemotherapy.

According to the manufacturer’s protocol, CTCs were enriched by lysis of erythrocytes and subsequent depletion of leukocytes. Briefly, red cell lysis buffer (v:v, 1:4) was firstly used to lyse the anticoagulant whole blood samples for 15min on ice. Then 200ml anti-CD45 coated magnetic beads were used to treat the cells for 30 min to deplete leucocytes. After that, CTCs were incubated with 10ml labeling buffer (folate-linked oligonucleotide) for 40min at room temperature. The cells were then washed 3 times with 1ml wash buffer at 500g. Finally, the cells were treated with 120ml stripping buffer to remove the ligand-oligonucleotide conjugates. The supernatant were collected by centrifugation and neutralized by 24ml neutralization buffer for further PCR analysis. Real time quantitative polymerase chain reaction was performed using the CytoploRare^®^ circulating lung cancer cell kit on ABI StepOne™ system (Life technologies). Two and half microliters of the prepared samples were added into a 25ml PCR reaction system following the manufacturer’s instruction manual. The PCR reaction conditions were as follows: denaturation at 95°C for 2 min, annealing at 40°C for 30 s, extension at 72°C for 30 s, then cooling at 8°C for 5 min; 40 cycles of denaturation at 95°C for 10 s, annealing at 35°C for 30 s, and extension at 72°Cfor 10 s. A serial of standards containing oligonucleotides (10^-14^ to 10^-9^M, corresponding to 2 to 2x10^5^ CTC units/3 ml blood) were used for CTC quantification. All patients’ samples were tested in duplicates with 6 standards and 3 quality controls. Following the manufacturer’s protocol, the mean intraassay variance (the maximum difference between duplicates) should be < 0.5 threshold cycle for the standards and quality controls, and < 1 threshold cycle for tested samples (11).

### Drug Susceptibility Predicted by CTCs Primary Cultured *In Vitro*

In this study, the patients resistant to at least the first-line chemotherapy (etoposide plus cisplatin or carboplatin) were enrolled. CTCs collection and the drug susceptibility tests were done by Polaris Biology in China. About 6 chemotherapeutic agents per patient were tested based on their previous medication histories if the numbers of CTCs collected were enough. Then these patients were treated with the highly sensitive chemotherapeutic drugs according to the test results.

**Figure 1:**
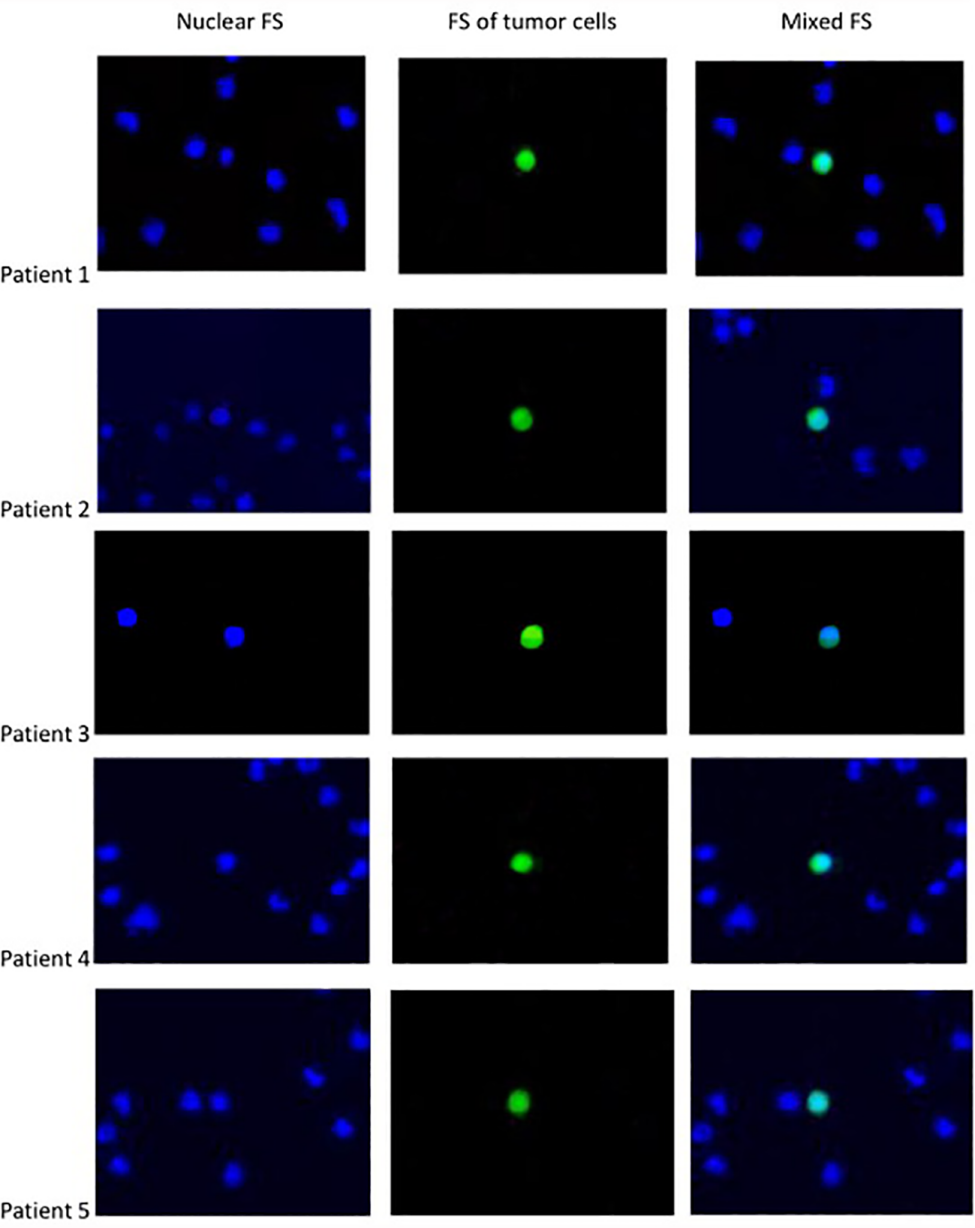
The fluorescent staining (FS) OF CTC.

7.5ml of peripheral blood was collected in either EDTA or Cell-Free DNA BCT^®^ tubes (Streck, USA) and processed within 24h, respectively. Next, red blood cell (RBC) lysis buffer was used to treat the prepared whole blood and the nucleated cell fraction was recovered. The nucleated cells were suspended in the custom ClearCell resuspension buffer, and loaded on the ClearCell FX^®^ System(Clearbridge BioMedics, Singapore). A new CTChip was loaded on the machine and the automated protocol was run. Within an hour, the enriched CTCs were collected in a 15ml centrifuge tube in suspension format and seamlessly integrated into downstream assays. After the enrichment, the system ran a cleaning cycle to avoid cross-contamination between samples. Because of the fast metabolic rate, cancer cells can rapidly absorb glucose, which has become the basic detection principle of PET-CT. So, PET-CT was used to identify and confirmed the CTCs (11). CTCs were then transferred into a 1.5mL tube, and washed three times using 1 × PBS (with 1% penicillin and streptomycin), and then transferred into ultra-low attachment 96-well plate for short-time expansion (2-4 days). Cell viability was assessed using the eBioscience™ Indo-1 AM Calcium SensorDye (Thermo Fisher) system. Cultured CTCs were incubated with 2 umol/L calcium dye system. With this dye and drug combination, viable CTCs are shown green and dead cells are dark (**Figure 1**). Imaging was performed with the NIKONE-C1confocal microscope system.

### Statistical Analyses

The statistical analysis about the correlation between changes in CTC counts and the responses to therapy was performed using IBM SPSS Statistics 23 software. The statistical significance analysis was calculated using chi-square test.

## Results

### Patient Characteristics

In the part of retrospective study, a total of 99 patients with unresectable SCLC received platinum-based doublet chemotherapy in the first-line setting. The mean age at diagnosis was 63.99 ± 8.81 years, 92% were male, and 68.9% had ES diseases. The median PFS was 9.83 months and the ORR was 74.7%, including 3 cases with CR, 62 cases with PR, 10 cases with SD, 12 cases with PD, and 12 cases intolerant to chemotherapy or without evaluation. A total of 36 cases after progression on the first-line therapy went on to the second-line chemotherapy and 11 cases after progression on the second-line therapy went on to the third-line chemotherapy. Baseline patient characteristics are presented in **Table 1**.

**Table 1:** Patient characteristics.

Variables	Mean
**Age (years)**	63.99 ± 8.81
**Sex**	
Male	91
Female	8
**Clinical stage**	
LD	31
ED	68
**Response to first-line therapy**	
CR	3
PR	62
SD	10
PD	12
Intolerant or without evaluation	12

### Efficacy of Later-Line Chemotherapy

In the second-line setting, 36 (36.3%) patients received Irinotecan followed by platinum-based chemotherapy. For all the second-line patients, the median PFS was 2.6 months, with only 2.8% surviving for more than a year. Within the third-line setting, 11 (11.1%) patients received chemotherapy and the others received BSC. Among actively treated patients, taxane-based single-agent regimens were the most commonly used regimens followed by Irinotecan. Only 1 patient was treated with immunotherapy across all lines of therapies. The average duration of chemotherapy was 2.4 months in the third-line setting and no one responded to chemotherapy (**Table 2**). Among the patients receiving BSC, more than half of them were classified as treatment-eligible to receive active treatments as determined by their ECOG status or duration of follow-up available. So new drugs or new methods to test the susceptibility of old drugs for individual SCLC patients are urgently needed.

**Table 2:** Response to chemotherapy in SCLC patients.

	Number	ORR	PFS (months)
First-line	99	74.7% (87 cases can be evaluated)	9.83
Second-line	36	11.1%	2.6
Third-line	11	0	2.4

### Correlation Between Change of CTC Counts and Treatment Response

To evaluate the change of CTC counts as a potential marker for monitoring therapy response, fourteen patients with SCLC in first-line setting were screened for CTCs counts test within one week before and after two cycles of chemotherapy, and then the patients were grouped into responsive, stable, and progressive disease based on therapeutic efficacy according to RECIST criteria 1.1. The results showed that the patients with responsive diseases had reduced CTC counts with a median decrease of 6.96 CTCs, and the patients with stable diseases had a median decrease of 3.34 CTCs, whereas the patients with progressive diseases got a median increase of 13.05 CTCs after chemotherapy (**Table 3**), the response of therapy was significantly related with the change of CTCs counts, *p<0.001*.

**Table 3:** Changes of CTC counts correlated with response of chemotherapy in SCLC patients.

Patient	Response	CTC counts		Difference
		Before therapy	After therapy	
1	PR	25.51	19.4	-6.11
2	PR	26.57	18.76	-7.81
3	SD	18.84	15.07	-3.77
4	SD	14.24	11.33	-2.91
5	PD	9	18.46	9.46
6	PD	14.98	39.21	24.23
7	PD	7.99	13.33	5.34
8	PD	9.48	15.96	6.48
9	PD	15.27	21.92	6.65
10	PD	27.75	35.58	20.83
11	PD	18.83	50.66	7.83
12	PD	6.31	27.14	31.83
13	PD	10.12	19.52	9.4
14	PD	15.07	23.52	8.45

### Relevance Between Efficacy and Drug Susceptibility of CTCs Cultured *In Vitro*

To evaluate the clinical value of this drug susceptibility platform using CTCs primary cultured *in vitro*, ten never used chemotherapeutic agents were tested for their sensitivities in five patients with SCLC after their second-line chemotherapy. The test reports are shown in **Table 4** (not all of ten drugs were detected in every patient because some patients had not enough CTCs). Patient 1 was highly sensitive to Docetaxel and Cisplatin, patient 2 to Docetaxel, patient 3 to Gemcitabine, Nedaplatin, Docetaxel, and Vinorelbine, patient 4 to Vinorelbine and Albumin paclitaxel, and patient 5 to Docetaxel. Four patients were treated with the highly sensitive drugs according to these results, but their disease progressed after two cycles of chemotherapy. The patient 5 didn’t receive chemotherapy because of his quick disease progression and the poor ECOG status. These results showed that the drug susceptibility test of CTCs primary cultured *in vitro* may not have distinct effect on clinical efficacy of patients with SCLC.

**Table 4:** Drug susceptibility test results.

Drug	Patient 1	Patient 2	Patient 3	Patient 4	Patient 5
Docetaxel	H	H	H	R	H
Vinorelbine	R	L	H	H	
Gemcitabine	R	L	H	R	L
Paclitaxel			L		
Albumin paclitaxel				H	
Cisplatin	H				
Nedaplatin		R	H		L
Luoplatinum	M	M	M	M	
Pemetrexed		M			
Irinotecan					M

*H, Highly sensitive; M, Moderately sensitive; L, Low sensitivity; R, Resistance.

## Discussion

In our study there are 99 patients with unresectable SCLC received platinum-based doublet chemotherapy in the first-line setting and the ORR was 74.7%. Due to the inaccessibility of topotecan and irinotecan was not used in first-line therapy, the patients with SCLC in our hospital received the treatment of irinotecan in second-line setting. Among them, 36 patients received Irinotecan treatment in the second-line setting, but the median PFS was only 2.6 months, and only 2.8% surviving for more than one year. In the third-line setting, 11 patients received chemotherapy, but no one responded. However, in the patients receiving BSC, more than half were classified as treatment-eligible to receive active treatments. So this study hopes to find some drugs sensitive to patients. Finally, we have shown that the culture of CTCs *in vitro* provides an opportunity to study patterns of drug susceptibility that is unique to an individual tumor although this technique is not well developed in patients with SCLC now.

CTCs circulating in the peripheral blood, with their role as a “tumor liquid biopsy”, provide convenient access to all disease sites. It is conceivable that detecting and analyzing CTCs will provide insightful information in assessing the disease status and monitoring the response of anticancer drugs. However, identifying CTCs in patient blood samples is technically challenging due to the extremely low abundance of CTCs among a large number of hematologic cells. The size of circulating tumor cells (~15-20 um) is significantly different from that of red blood cells (~8 um) and white blood cells (~8-15 um). In addition, CTC has higher nuclear-cytoplasmic ratio and irregular cell morphology. These characteristics make CTCs different from other cells in fluid characteristics and flow rate (12). Researchers made great efforts to screen and separate them, because they have the potential to be used in a number of ways, for example, patient cohorts could be selected based on the drug sensitivity pre-screening, alternatively, acquired resistance to chemotherapy can be monitored throughout the progress of clinical trials.

Most PFS of patients in the CTC counts study were less than 2 months, and their OS were also very poor, and due to the limited time and fund, we can’t recruit more patients and collect more samples in this study. But many studies have proved the correlation between the change of CTC counts and the response of therapy, so we didn’t do the statistical evaluation. However, the technology of folate receptor targeted PCR can’t get the intact and viable CTCs. There are two modes of sorting to isolate CTCs till now. One is a negative selection mode (negCTC-iChip), in which the blood sample is depleted of leukocytes by immunomagnetically targeting both the common leukocyte antigen CD45 and the granulocyte marker CD15 (13). The other is a novel platform presented in this paper for prediction of efficacy of cancer drugs based on CTCs primary cultured *in vitro*. The ClearCell^®^ FX System, a label-free microfluidics technology that utilizes Dean Flow Fractionation principle in a spiral microfluidics system to separate the larger CTCs from smaller blood cells, driven by the CTChip^®^ FR biochip, is one of the world’s first automated cell retrieval systems that can enrich wholly intact and viable CTCs from blood in a relatively short time. The automated system performs a single-step CTCs isolation and retrieval and collects the enriched CTCs in suspension format, achieving extremely high recovery rates. CTCs with high activity and no damage can be naturally separated from other cells in the sample based on the difference of flow velocity (12). After that, the chemotherapeutic drugs susceptibility was detected in primary cultured CTCs *in vitro* and then the patients were treated according to the results obtained, but we didn’t get the expected treatment response.

Although our present findings indicate that the drug susceptibility test of the CTCs cultured *in vitro* may have little effect on the clinical efficacy of SCLC, it still needs to be validated in larger studies. The other possible reason for this result may be that SCLC is a highly aggressive malignant disease and resistant to all the old drugs in our tests or that the tumor microenvironment has some influences on the drug efficacy. Krohn et al. found that many tumors acquired drug resistance and their neuroendocrine differentiation was lost during epithelial-mesenchymal transition (EMT) of SCLC cells, indicating that drug resistance is one characteristic of EMT (14). Hamilton et al. obtained a panel of SCLC CTC cell line from patients with relapsing disease, which share a primarily epithelial phenotype with high expression of EpCAM, absent phosphorylation of β-catenin and background levels of Snail (15). Maybe that’s why in our study the SCLC-CTCs sensitive to some drugs but resistant *in vivo*.

## Data Availability Statement

The original contributions presented in the study are included in the article/supplementary material. Further inquiries can be directed to the corresponding author.

## Ethics Statement

The studies involving human participants were reviewed and approved by The Ethics Committee of Shanghai Pulmonary Hospital (approval no. K19-137). The patients/participants provided their written informed consent to participate in this study.

## Author Contributions

JL, YJ, ZC and CS: substantial contributions to the conception or design, acquisition, analysis, or interpretation of data, critical revision for important intellectual content, final approval of the version to be published. DZ and LM: coordinate and to be accountable for all aspects of the work in ensuring that questions related to the accuracy or integrity of any part of the work. All authors contributed to the article and approved the submitted version.

## Funding

Science and Technology Commission of Shanghai Municipality (17401932400; 19401930800); National Natural Science Foundation of China (81207106); Shanghai Pulmonary Hospital (fkgg1807).

## Conflict of Interest

The authors declare that the research was conducted in the absence of any commercial or financial relationships that could be construed as a potential conflict of interest.

## References

[1] Sung H Ferlay J Siegel RL Laversanne M Soerjomataram I Jemal A. Global Cancer Statistics 2020: GLOBOCAN Estimates of Incidence and Mortality Worldwide for 36 Cancers in 185 Countries. CA Cancer J Clin (2021). 10.3322/caac.21660 33538338

[2] Dingemans AC Früh M Ardizzoni A Besse B Faivre-Finn C Hendriks LE. Small-Cell Lung Cancer: ESMO Clinical Practice Guidelines for Diagnosis, Treatment and Follow-Up☆. Ann Oncol (2021). 10.1016/j.annonc.2021.03.207 PMC9464246 33864941

[3] National Comprehensive Cancer Network. Small Cell Lung Cancer (V2.2018). Fort Washington, PA. Available at: https://www.nccn.org/professionals/physician_gls/pdf/sclc.pdf (Accessed 16 February 2018).

[4] Facchinetti F Di Maio M Tiseo M. Adding PD-1/PD-L1 Inhibitors to Chemotherapy for the First-Line Treatment of Extensive Stage Small Cell Lung Cancer (Sclc): A Meta-Analysis of Randomized Trials. Cancers (Basel) (2020) 12(9). 10.3390/cancers12092645 PMC7565587 32947924

[5] Horn L Mansfield AS Szczęsna A Havel L Krzakowski M Hochmair M. First-Line Atezolizumab Plus Chemotherapy in Extensive-Stage Small-Cell Lung Cancer. N Engl J Med (2018) 379(23):2220–96. 10.1056/NEJMoa1809064 30280641

[6] Noda K Nishiwaki Y Kawahara M Negoro S Sugiura T Yokoyama A. Japan Clinical Oncology Group.Irinotecan Plus Cisplatin Compared With Etoposide Plus Cisplatin for Extensive Small-Cell Lung Cancer. N Engl J Med (2002) 346:85–91. 10.1056/NEJMoa003034 11784874

[7] Lara PN Jr Natale R Crowley J Lenz HJ Redman MW Carleton JE. Phase III Trial Ofirinotecan/Cisplatin Compared With Etoposide/Cisplatin in Extensive-Stagesmall-Cell Lung Cancer: Clinical and Pharmacogenomic Results From SWOG S0124. J Clin Oncol (2009) 27:2530–5. 10.1200/JCO.2008.20.1061 PMC2684855 19349543

[8] Hanna N Bunn PA Jr Langer C Einhorn L Guthrie T Beck T. Randomized PhaseIII Trial Comparing Irinotecan/Cisplatin With Etoposide/Cisplatin in Patients With Previously Untreated Extensive-Stage Disease Small-Cell Lung Cancer. J Clin Oncol (2006) 24:2038–43. 10.1200/JCO.2005.04.8595 16648503

[9] Hou JM Krebs MG Lancashire L Lancashire L Sloane R Backen A. Clinical Significance and Molecular Characteristics of Circulating Tumor Cells and Circulating Tumor Microemboli in Patients With Small-Cell Lung Cancer. J Clin Oncol (2012) 30:525–32. 10.1200/JCO.2010.33.3716 22253462

[10] Huang CH Wick JA Sittampalam GS Nirmalanandhan VS Ganti AK Neupane PC. A Multicenter Pilot Study Examining the Role of Circulating Tumor Cells as a Blood-Based Tumor Marker in Patients With Extensive Small-Cell Lung Cancer. Front Oncol (2014) 4:271. 10.3389/fonc.2014.00271 25353007 PMC4196518

[11] Lou J Ben S Yang G Liang X Wang X Ni S. Quantification of Rare Circulating Tumor Cells in Non-Small Cell Lung Cancer by Ligand-Targeted PCR. PloS One (2013) 8:e80458. 10.1371/journal.pone.0080458 24324600 PMC3855610

[12] Lee Y Guan G Bhagat AA. Clearcell® FX, a Label-Free Microfluidics Technology for Enrichment of Viable Circulating Tumor Cells. Cytometry A (2018) 93:1251–54. 10.1002/cyto.a.23507 30080307

[13] Yu M Bardia A Aceto N Bersani F Madden MW Donaldson MC. Cancer Therapy. Ex Vivo Culture of Circulating Breast Tumor Cells for Individualized Testing of Drug Susceptibility. Science (2014) 345(6193):216–20. 10.1126/science.1253533 PMC4358808 25013076

[14] Krohn A Ahrens T Yalcin A Plönes T Wehrle J Taromi S. Tumor Cell Heterogeneity in Small Cell Lung Cancer (SCLC): Phenotypical and Functional Differences Associated With Epithelial-Mesenchymal Transition (EMT) and DNA Methylation Changes. PloS One (2014) 9(6):e100249. 10.1371/journal.pone.0100249 24959847 PMC4069054

[15] Hamilton G Rath B. Mesenchymal-Epithelial Transition and Circulating Tumor Cells in Small Cell Lung Cancer. Adv Exp Med Biol (2017) 994:229–45. 10.1007/978-3-319-55947-6_12 28560677

